# Association between stent length and number and the risk of in-stent restenosis in patients after percutaneous coronary intervention: a systematic review and meta-analysis

**DOI:** 10.3389/fcvm.2025.1673698

**Published:** 2025-10-09

**Authors:** Chanchun Cai, Lingfen Lu, Xiaojun Ji, Zhongping Shi

**Affiliations:** ^1^ Department of Intervention Center, Wenzhou Central Hospital, Wenzhou, Zhejiang, China; ^2^ Department of Cardiovascular Medicine, Wenzhou Central Hospital, Wenzhou, Zhejiang, China

**Keywords:** stent length, stent number, stent restenosis, percutaneous coronary intervention, meta-analysis, coronary artery disease

## Abstract

**Background:**

The association of stent length and number with the risk of in-stent restenosis (ISR) following percutaneous coronary intervention (PCI) in patients with coronary artery disease (CAD) has been widely reported, yet findings remain inconsistent across studies. To clarify this relationship, we conducted a meta-analysis of observational studies evaluating the impact of stent length and number on ISR risk after PCI in CAD patients.

**Methods:**

Case-control studies addressing stent length, stent number, and ISR after PCI in CAD patients were systematically searched in electronic databases including VIP, Wanfang, CNKI, Chinese Biomedical Literature Database, PubMed, Web of Science and the Cochrane Library from inception until June 2025. The Risk Of Bias In Non-randomized Studies of Interventions (ROBINS-I) tool was used to assess study quality. Pooled odds ratios (ORs) and 95% confidence intervals (CIs) were calculated using a random-effects model. All analyses were performed with Review Manager version 5.4.

**Results:**

Eighteen studies involving 6,585 participants were included. Meta-analyses indicated that both stent length [OR = 1.05, 95% CI (1.04, 1.07), *P* < 0.00001] and stent number [OR = 3.01, 95% CI (1.97, 4.59), *P* < 0.00001] were significant risk factors for ISR after PCI in CAD patients.

**Conclusion:**

This meta-analysis supports the conclusion that stent length and number are associated with an increased risk of ISR after PCI in CAD patients. However, given the limited number and moderate quality of the included studies, these findings should be interpreted with caution and validated by further high-quality research.

## Introduction

Coronary artery disease (CAD) has become one of the leading causes of death worldwide (1–3). Percutaneous coronary intervention (PCI), a key treatment for CAD, enables rapid vascular reperfusion by dilating the coronary lumen, preserves myocardial tissue, and significantly reduces patient mortality (4). However, PCI is often associated with various complications, among which in-stent restenosis (ISR) accounts for approximately 10% of cases (5). ISR can lead to symptom recurrence and increase the risk of adverse cardiovascular events (6), adversely affecting patients’ quality of life and often necessitating repeat interventions.

Currently, drug-eluting stents (DES) and drug-coated balloons (DCB) are recommended for treating ISR (7, 8). Among available options—including plain balloon angioplasty, bare-metal stents (BMS), DES, DCB, and cutting balloons—the cobalt-chromium everolimus-eluting stent (EES) has been shown to be the most effective strategy for ISR management (8). Nevertheless, the search for optimal ISR therapies continues. Bioresorbable vascular scaffolds (BVS), which offer a “leave-nothing-behind” approach, have attracted attention in this context. BVS provide short- to mid-term radial strength comparable to BMS and DES, while eventually being fully absorbed in the body (9). Theoretically, this allows restoration of native vessel elasticity and endothelial function, reduces late stent malapposition, and prevents very late stent thrombosis caused by incomplete endothelialization of metallic stents (10). However, some studies indicate that biodegradable scaffolds may be associated with higher rates of adverse cardiovascular events compared with BMS, particularly target vessel myocardial infarction and scaffold thrombosis (11, 12). Therefore, understanding the risk factors, mechanisms, treatment, and prevention of coronary ISR remains critically important.

The mechanism of ISR is not yet fully elucidated. Studies suggest that neointimal hyperplasia within 3–6 months after PCI is a major contributor to ISR (13). Other research indicates that the severity of ISR is closely associated with biochemical markers such as serum lipoproteins, uric acid, and high-sensitivity C-reactive protein, with dyslipidemia also playing a potential role (4, 14, 15). In recent years, risk factors for ISR after PCI in CAD patients have been widely investigated (16–21). However, evidence regarding the impact of stent length and number on ISR remains inconsistent and warrants further clarification. Thus, this study conducted a meta-analysis of published studies evaluating the association of stent length and number with ISR risk after PCI, aiming to provide more robust evidence for clinical decision-making.

## Materials and methods

### Search strategy

Case-control studies investigating the association of stent length and number with ISR after PCI in patients with CAD were retrieved via computerized searches of the following databases: VIP, Wanfang, CNKI, Chinese Biomedical Literature Database, PubMed, Web of Science and the Cochrane Library. The search period spanned from the inception of each database to June 2025. A combination of subject headings and free-text terms was employed, with adjustments made according to the specific features of each database. References of included studies were also manually screened to identify additional relevant publications. Search keywords included: “coronary artery disease”, “in stent restenosis”, “percutaneous coronary intervention”, “risk factor”, etc. The detailed search strategy is provided in Supplementary Table S1.

### Inclusion and exclusion criteria

Inclusion criteria were as follows:

1. Participant: CAD patients who underwent PCI, with the case group defined as those who developed ISR and the control group as those without ISR;
2. Exposure: Stent number and/or stent length;
3. Outcomes: ISR defined as ≥50% diameter stenosis on coronary angiography within the stent or within 5 mm of its proximal or distal edge, as referenced to the adjacent normal vessel segment (22);
4. Study design: Case-control studies.

Exclusion criteria included:

1. Case reports, reviews, or animal studies;
2. Studies with incomplete data and where authors could not be contacted for additional information;
3. Duplicate publications.

### Data extraction

Two evaluators independently screened literature, extracted data and cross-checked the results. Disagreements were resolved through discussion or by consultation with a third reviewer. When necessary, corresponding authors were contacted to obtain missing data. The following information was extracted:

① Basic study characteristics: first author, publication year, country, etc.; ② baseline characteristics of the study population; ③ key elements related to risk of bias assessment; ④ outcome measures and relevant effect estimates.

### Risk of bias assessment

The risk of bias in the included observational studies was evaluated independently by two reviewers using the Risk Of Bias In Non-randomized Studies of Interventions (ROBINS-I) tool. The assessment covered seven domains: bias due to confounding, participant selection, classification of interventions, deviations from intended interventions, missing data, outcome measurement, and selective reporting. Each domain was judged as “low risk,” “moderate risk,” “high risk,”, “critical risk”, and as “no information.”

### Statistical analysis

Meta-analysis was conducted using RevMan version 5.4 software. Odds ratios (ORs) with 95% confidence intervals (CIs) were calculated for dichotomous outcomes. Heterogeneity among studies was assessed using the *χ*^2^ test (significance level set at *α* = 0.10) and quantified with the *I*2 statistic. An *I*^2^ < 50% and *P* > 0.10 indicated acceptable heterogeneity, in which case a fixed-effects model was applied; otherwise, a random-effects model was used. Given expected variations in study populations, treatment protocols, and follow-up durations, a random-effects model was preferred for its ability to account for clinical and methodological diversity and to provide more generalized estimates (23). Sensitivity analysis was performed by sequentially excluding each study to test the robustness of the results. Publication bias was assessed using funnel plots, Begg's Test and Egger's test.

## Results

### Search results

**Figure 1:**
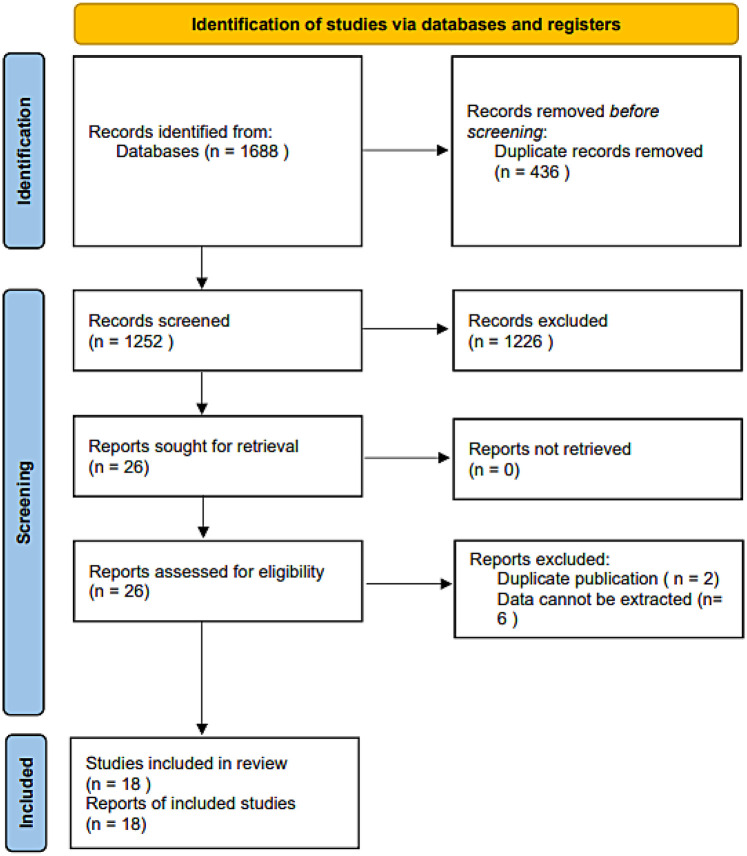
Flow diagram for the eligible study selection process.

The initial database search identified 1,688 relevant records. After removing 436 duplicates, 1,226 records were excluded based on title and abstract screening. Following a full-text review of the remaining articles, eight were excluded for not meeting the eligibility criteria. Ultimately, 18 studies (17–19, 24–38) were included in the meta-analysis (Figure 1).

### Characteristics of the eligible studies

All 18 included studies (17–19, 24–38) were case-control designs, involving a total of 6,585 patients (1,431 cases with ISR and 5,154 controls). Two risk factors were examined across these studies: stent length was reported in twelve articles (17, 19, 24, 25, 27, 28, 30, 31, 33–35, 38), and stent number was analyzed in ten articles (17–19, 26, 28, 29, 32, 36–38). The basic characteristics of the included studies are summarized in Table 1. Based on the ROBINS-I tool, the risk of bias assessment indicated that thirteen studies had a moderate risk of bias, while five studies were judged to have a low risk of bias (Table 2).

**Table 1:** Characteristics of the studies included in meta analysis.

Study	Study design	Region	Age (Years)		Sample size (*n*)		Risk factors
			Case	Control	Case	Control	
Li et al. (17)	Case-control study	China	66.71 ± 9.65	65.63 ± 11.15	62	279	①②
Xu et al. (18)	Case-control study	China	62.3 ± 9.1	60.4 ± 10.3	95	517	②
Yildiz et al. (24)	Case-control study	Turkey	61.9 ± 11.0	61.3 ± 10.7	131	138	①
Zhang et al. (45)	Case-control study	China	68.23 ± 8.67	69.15 ± 9.04	138	212	①②
Liu et al. (28)	Case-control study	China	—	—	47	76	①②
Tang et al. (31)	Case-control study	China	59.23 ± 11.52	58.94 ± 14.62	36	138	①
Zhang et al. (34)	Case-control study	China	58.98 ± 7.89	59.32 ± 9.21	51	442	①
Zhang et al. (35)	Case-control study	China	69.14 ± 8.57	66.35 ± 9.21	38	82	①
Zhu et al. (38)	Case-control study	China	—	—	145	1,013	①②
Li et al. (27)	Case-control study	China	—	—	90	110	①
Yang (33)	Case-control study	China	60.90 ± 6.91	60.33 ± 9.31	63	70	①
Pan (30)	Case-control study	China	66 ± 9	65 ± 10	258	262	①
Zhao and Zhang (36)	Case-control study	China	57.31 ± 8.45	57.31 ± 8.45	45	200	②
Deng et al. (25)	Case-control study	China	63.53 ± 11.81	62.21 ± 11.06	89	1,253	①
Zheng et al. (37)	Case-control study	China	59.4 ± 9.4	59.1 ± 9.0	21	105	②
Lu et al. (29)	Case-control study	China	60.17 ± 9.47	58.27 ± 10.43	87	68	②
Wei et al. (32)	Case-control study	China	68.1 ± 16.6	70.1 ± 17.5	4	46	②
Li et al. (26)	Case-control study	China	60.7 ± 11.5	61.9 ± 11.6	31	143	②

①Stent length; ②Stent number.

**Table 2:** ROBINS-I assessment of study bias for included studies.

Study	D1	D2	D3	D4	D5	D6	D7	Overall risk of bias
Li et al. (17)	Moderate	Low	Low	Low	Low	Low	Low	Moderate
Xu et al. (18)	Moderate	Low	Low	Low	Low	Low	Low	Moderate
Yildiz et al. (24)	Low	Low	Moderate	Low	Low	Low	Low	Moderate
Zhang et al. (45)	Moderate	Low	Low	Low	Low	Low	Low	Moderate
Liu et al. (28)	Low	Low	Low	Low	Moderate	Low	Low	Moderate
Tang et al. (31)	Low	Low	Moderate	Low	Low	Low	Low	Moderate
Zhang et al. (34)	Low	Low	Low	Low	Low	Low	Low	Low
Zhang et al. (35)	Low	Low	Low	Moderate	Low	Low	Low	Moderate
Zhu et al. (38)	Low	Low	Moderate	Low	Moderate	Low	Low	Moderate
Li et al. (27)	Low	Low	Low	Low	Low	Low	Low	Low
Yang (33)	Low	Moderate	Low	Low	Moderate	Low	Low	Moderate
Pan (30)	Low	Low	Low	Low	Low	Low	Low	Low
Zhao and Zhang (36)	Low	Low	Low	Moderate	Moderate	Low	Low	Moderate
Deng et al. (25)	Low	Low	Moderate	Low	Low	Low	Low	Moderate
Zheng et al. (37)	Low	Moderate	Low	Low	Low	Low	Low	Moderate
Lu et al. (29)	Low	Low	Low	Low	Low	Low	Low	Low
Wei et al. (32)	Moderate	Low	Low	Low	Low	Low	Low	Moderate
Li et al. (26)	Low	Low	Low	Low	Low	Low	Low	Low

Domains:

D1: Bias due to confounding.

D2: Bias in selection of participants.

D3: Bias in classification of exposures.

D4: Bias due to deviations from intended exposures.

D5: Bias due to missing data.

D6: Bias in measurement of outcomes.

D7: Bias in selection of the reported result.

### Meta-analysis results

**Figure 2:**
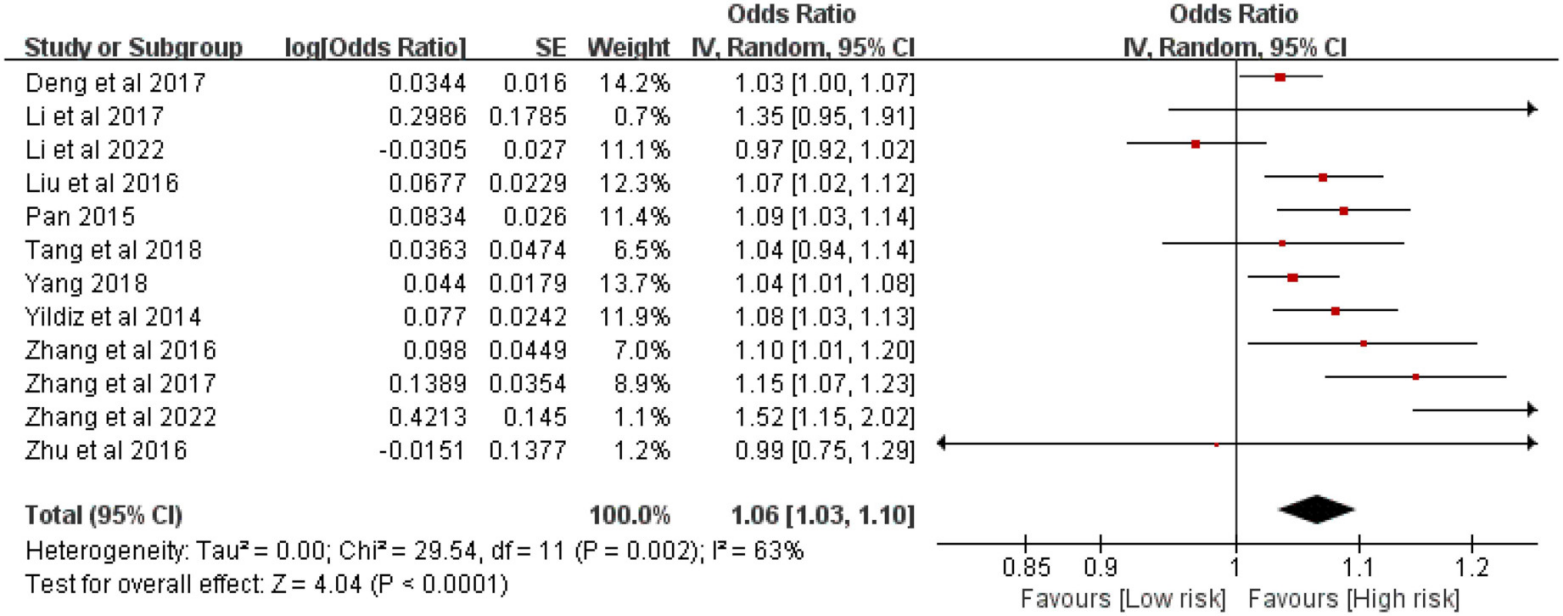
Forest plot of the association between stent length and the risk of in stent restenosis in patients after percutaneous coronary intervention.

Twelve studies involving 5,223 patients evaluated the association between stent length and ISR risk after PCI in CAD patients. Heterogeneity was significant (*P* = 0.002, *I*^2^ = 63%). The random-effects meta-analysis showed that longer stent length was significantly associated with an increased risk of ISR [OR = 1.05, 95% CI (1.04, 1.07), *P* < 0.00001] (Figure 2).

**Figure 3:**
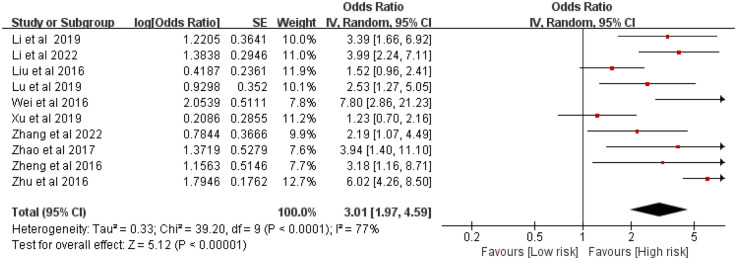
Forest plot of the association between stent number and the risk of in stent restenosis in patients after percutaneous coronary intervention.

Ten studies comprising 3,334 patients examined the relationship between the number of stents and ISR risk. Considerable heterogeneity was observed (*P* < 0.0001, *I*^2^ = 77%). The meta-analysis indicated that a greater number of stents significantly elevated the risk of ISR [OR = 3.01, 95% CI (1.97, 4.59), *P* < 0.00001] (Figure 3).

### Sensitivity analysis

**Figure 4:**
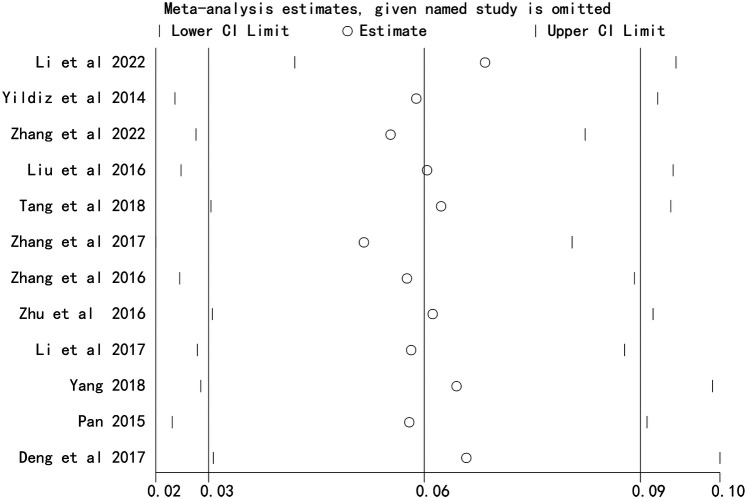
Sensitivity analysis of the association between stent length and the risk of in stent restenosis in patients after percutaneous coronary intervention.

**Figure 5:**
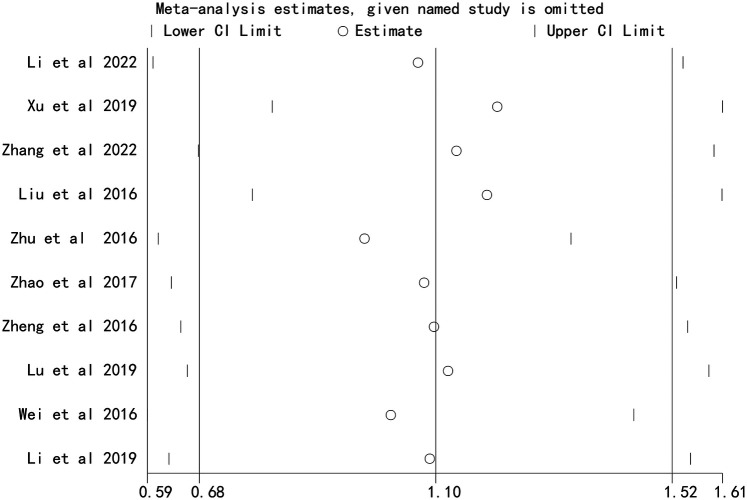
Sensitivity analysis of the association between stent number and the risk of in stent restenosis in patients after percutaneous coronary intervention.

Sensitivity analysis, performed by sequentially excluding each study, demonstrated that the overall effect estimates remained stable, indicating that the results were not driven by any single study (Figures 4 and 5).

### Publication bias

**Figure 6:**
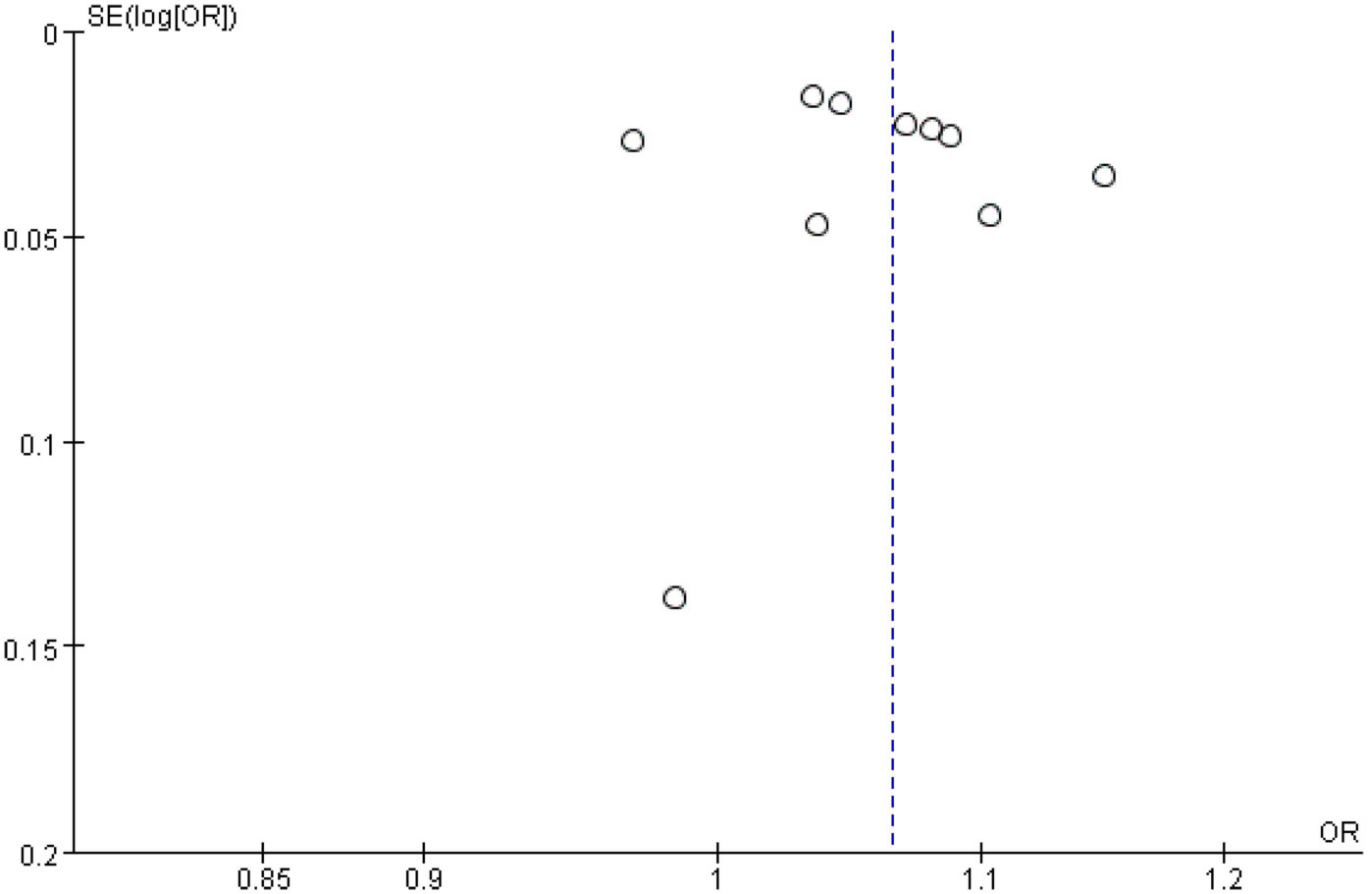
Funnel plot of sensitivity analysis of the association between stent length and the risk of in stent restenosis in patients after percutaneous coronary intervention.

**Figure 7:**
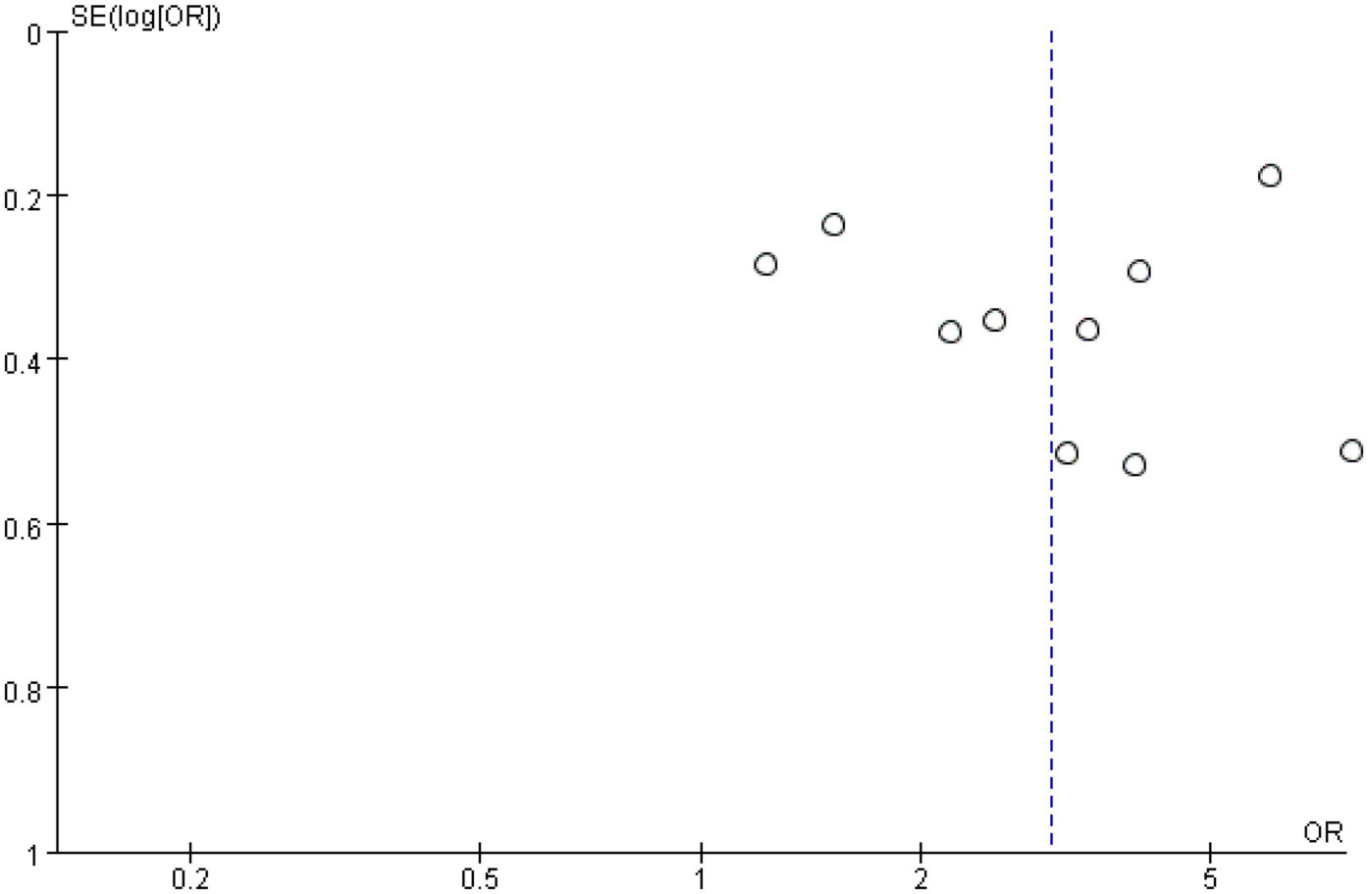
Funnel plot of the association between stent number and the risk of in stent restenosis in patients after percutaneous coronary intervention.

Funnel plots for stent length and stent number were generally symmetrical, suggesting a low likelihood of publication bias (Figures 6 and 7). This was further supported by Begg's Test and Egger's test, which showed no significant evidence of publication bias (Table 3).

**Table 3:** Publication bias analysis.

Outcomes	Begg's test (*P*-value)	Egger's test (*P*-value)
Stent length	0.386	0.543
Stent number	0.174	0.065

## Discussion

We conducted a meta-analysis to evaluate the association of stent length and number with the risk of ISR after PCI in CAD patients. Based on a comprehensive analysis of eighteen observational studies, we found that both longer stent length and a greater number of stents were significantly associated with an increased risk of ISR, with stent number showing a particularly strong effect. These results underscore the importance of stent characteristics in determining ISR risk after PCI.

The pathogenesis of ISR involves a complex pathological process, including smooth muscle cell proliferation and migration, sustained inflammatory responses, and the development of in-stent neoatherosclerosis (ISNA) (39–41). During PCI, stent length and number are selected based on lesion morphology, angulation, and side-branch involvement (42, 43). Longer stents can increase procedural complexity, require more balloon inflations, and exacerbate vascular injury and inflammation, thereby elevating ISR risk (4, 44). In practice, to ensure complete coverage of dissections or diseased segments and maintain lumen patency, stents are typically extended several millimeters beyond the angiographic margins of the lesion (45). However, longer stents may cause more extensive vascular damage and induce unfavorable hemodynamic changes, creating conditions conducive to ISR (46, 47). Hong et al. (48) identified stent length ≥40 mm as an independent risk factor for ISR after PCI in CAD patients. Extended stent length may intensify endothelial injury, while also negatively affecting the natural healing process of blood vessels, thereby promoting the proliferation and migration of smooth muscle cells and ultimately leading to the occurrence of restenosis (49, 50). Therefore, in clinical practice, selecting an appropriate stent length is critical, particularly in high-risk patients.

An increased number of stents was also strongly associated with ISR risk, likely due to the amplified biological response following multiple stent implantations (16). The presence of multiple stents introduces more foreign material and may alter local hemodynamics, delaying the healing and regeneration process of the blood vessels (43). Moreover, the contact surface of multiple stents is enlarged, which may enhance inflammatory activation and neointimal hyperplasia (51, 52). Li et al. (17) reported that patients with ISR received more stents than those without ISR. Overlapping stents can cause geometric interference, disturb laminar flow, and promote abnormal endothelial growth (53). In addition, implanting multiple stents increases vascular resistance and requires higher deployment pressures, which may aggravate endothelial injury and trigger platelet adhesion, contributing to ISR (36, 54). This risk is particularly pronounced in small-vessel PCI, where stent placement is more likely to cause intimal damage and subsequent restenosis (30, 35). Thus, minimizing the number of stents represents an important clinical strategy during PCI.

The development and progression of ISR are influenced by multiple factors, including stent type, diabetes mellitus, vessel diameter, clinical presentation (acute vs. chronic), bifurcation lesions, and lesion complexity. A deeper understanding of their interactions is essential for developing effective prevention and treatment strategies for ISR. Diabetes, bifurcation lesions, small vessel diameter, and complex lesions significantly increase ISR risk (55). In diabetic patients, upregulation of pro-inflammatory cytokines may intensify vascular inflammation and promote restenosis (56, 57). Longer lesion length is also associated with higher ISR risk; for every 10 mm increase in lesion length, the percent diameter stenosis rises by an absolute 7.7% (46). Vessel diameter is another key anatomical factor and a strong predictor of ISR after both bare-metal stent (BMS) and drug-eluting stent (DES) implantation (58). ISR risk was significantly higher with BMS than with DES (55). The incidence of coronary ISR in early balloon angioplasty exceeded 50%. The application of BMS reduced it to 20%–30%, and DES further lowered it to 5%–15% (59). Second-generation DES, with improved polymer coatings and drug-release kinetics, have achieved even lower restenosis rates (60). The studies included in our analysis span a considerable period, covering the evolution from BMS to first- and second-generation DES. This technological progress has substantially altered the mechanisms and incidence of restenosis. Second-generation DES, featuring enhanced stent platforms, biocompatible polymers, and antiproliferative drugs, significantly reduce ISR risk compared with BMS and first-generation DES. Advances in implantation techniques—such as routine intravascular imaging for optimal sizing and expansion, high-pressure post-dilation, and improved perioperative medication—have also contributed to lowering restenosis risk. As the included studies cover different eras, they naturally reflect this technological progression. Future studies should consider stratifying analyses by stent generation (especially comparing first- vs. second-generation DES) to better reflect contemporary practice. Patients undergoing complex PCI (long stents/multiple stents) may benefit from intensified antithrombotic regimens to reduce thrombotic events, though bleeding risks must be balanced. Oliva et al. (61) reported that P2Y12 inhibitor monotherapy offered improved safety regarding major bleeding compared with standard dual antiplatelet therapy (DAPT) in complex PCI patients, without increasing ischemic events. In fact, P2Y12 inhibitor monotherapy was associated with a lower risk of myocardial infarction than DAPT. Bioresorbable vascular scaffolds (BVS) avoid long-term foreign-body reactions but carry a higher risk of early scaffold thrombosis, partly due to vascular recoil and delayed endothelial healing. Biodegradable stents have potential advantages in reducing ISR (62). These stents can provide the necessary support and drug release, and then gradually degrade while promoting the self-repair ability of blood vessels. However, although biodegradable stents show promise in preventing ISR, further long-term follow-up studies are still needed to verify their safety and durability (63). Future research should aim to elucidate the interplay of these multifactorial processes to improve clinical management of ISR and enhance patient quality of life.

## Limitations of this study

Our meta-analysis offers robust evidence supporting the association of stent length and number with the risk of ISR. However, several limitations should be considered. First, the number of available studies was limited, particularly from certain geographical regions and diverse populations, which may affect the generalizability of our findings. Second, although no significant publication bias was detected, the potential influence of unpublished negative results cannot be entirely ruled out. Significant heterogeneity was observed in the meta-analysis, which may be attributed to variations in reported stent length and number, as well as differences in sample sizes across studies. These factors could affect the reliability of the conclusions.

## Conclusion

In summary, our meta-analysis confirms that stent length and number are significant risk factors for ISR after PCI in patients with CAD. These findings offer valuable insights for clinicians in stent selection and provide a basis for further research on stent design and treatment strategy optimization. Given the limitations related to sample size, study design, and heterogeneity, future high-quality studies are warranted to validate these results.

## Data Availability

The datasets presented in this study can be found in online repositories. The names of the repository/repositories and accession number(s) can be found in the article/Supplementary Material.
