# Supplementary material for: Association between stent length and number and the risk of in-stent restenosis in patients after percutaneous coronary intervention: a systematic review and meta-analysis
Source: Front Cardiovasc Med. 2025 Oct 9;12:1673698. doi: 10.3389/fcvm.2025.1673698 (PMC12546172; doi:10.3389/fcvm.2025.1673698)
Supplement: Supplementary file 1 [file Table1.docx]

| **Table S1.Pubmed search strategy** | |
| --- | --- |
| **Number** | **Search terms** |
| #1 | Cardiovascular Diseases[MeSH Terms] |
| #2 | Cardiovascular Disease[Title/Abstract] |
| #3 | Disease, Cardiovascular[Title/Abstract] |
| #4 | Heart Diseases[MeSH Terms] |
| #5 | Heart Disease[Title/Abstract] |
| #6 | Cardiac Disorders[Title/Abstract] |
| #7 | Cardiac Disorder[Title/Abstract] |
| #8 | Heart Disorders[Title/Abstract] |
| #9 | Heart Disorder[Title/Abstract] |
| #10 | Cardiac Diseases[Title/Abstract] |
| #11 | Cardiac Disease[Title/Abstract] |
| #12 | Coronary Artery Disease[MeSH Terms] |
| #13 | Artery Disease, Coronary[Title/Abstract] |
| #14 | Artery Diseases, Coronary[Title/Abstract] |
| #15 | Coronary Artery Diseases[Title/Abstract] |
| #16 | Coronary Arteriosclerosis[Title/Abstract] |
| #17 | Arterioscleroses, Coronary[Title/Abstract] |
| #18 | Coronary Arterioscleroses[Title/Abstract] |
| #19 | Arteriosclerosis, Coronary[Title/Abstract] |
| #20 | Atherosclerosis, Coronary[Title/Abstract] |
| #21 | Atheroscleroses, Coronary[Title/Abstract] |
| #22 | Coronary Atheroscleroses[Title/Abstract] |
| #23 | Coronary Atherosclerosis[Title/Abstract] |
| #24 | Left Main Coronary Artery Disease[Title/Abstract] |
| #25 | Left Main Coronary Disease[Title/Abstract] |
| #26 | Left Main Disease[Title/Abstract] |
| #27 | Left Main Diseases[Title/Abstract] |
| #28 | Coronary Disease[MeSH Terms] |
| #29 | Coronary Diseases[Title/Abstract] |
| #30 | Disease, Coronary[Title/Abstract] |
| #31 | Diseases, Coronary[Title/Abstract] |
| #32 | Coronary Heart Disease[Title/Abstract] |
| #33 | Coronary Heart Diseases[Title/Abstract] |
| #34 | Disease, Coronary Heart[Title/Abstract] |
| #35 | Diseases, Coronary Heart[Title/Abstract] |
| #36 | Heart Disease, Coronary[Title/Abstract] |
| #37 | Heart Diseases, Coronary[Title/Abstract] |
| #38 | Ischemic heart disease[Title/Abstract] |
| #39 | Myocardial infarction[Title/Abstract] |
| #40 | Stable angina[Title/Abstract] |
| #41 | Unstable angina[Title/Abstract] |
| #42 | Acute Coronary Syndrome[MeSH Terms] |
| #43 | Acute Coronary Syndromes[Title/Abstract] |
| #44 | Coronary Syndrome, Acute[Title/Abstract] |
| #45 | Coronary Syndromes, Acute[Title/Abstract] |
| #46 | Syndrome, Acute Coronary[Title/Abstract] |
| #47 | Syndromes, Acute Coronary[Title/Abstract] |
| #48 | OR/1-47 |
| #49 | Coronary Restenosis[MeSH Terms] |
| #50 | Coronary Restenoses[Title/Abstract] |
| #51 | Restenoses, Coronary[Title/Abstract] |
| #52 | Restenosis, Coronary[Title/Abstract] |
| #53 | In stent restenosis[Title/Abstract] |
| #54 | In-stent restenosis[Title/Abstract] |
| #55 | ISR[Title/Abstract] |
| #56 | Intrastent restenosis[Title/Abstract] |
| #57 | Restenosisin-stent[Title/Abstract] |
| #58 | OR/49-57 |
| #59 | Percutaneous Coronary Intervention[MeSH Terms] |
| #60 | Coronary Intervention, Percutaneous[Title/Abstract] |
| #61 | Coronary Interventions, Percutaneous[Title/Abstract] |
| #62 | Intervention, Percutaneous Coronary[Title/Abstract] |
| #63 | Interventions, Percutaneous Coronary[Title/Abstract] |
| #64 | Percutaneous Coronary Interventions[Title/Abstract] |
| #65 | Percutaneous Coronary Revascularization[Title/Abstract] |
| #66 | Coronary Revascularization, Percutaneous[Title/Abstract] |
| #67 | Coronary Revascularizations, Percutaneous[Title/Abstract] |
| #68 | Percutaneous Coronary Revascularizations[Title/Abstract] |
| #69 | Revascularization, Percutaneous Coronary[Title/Abstract] |
| #70 | Revascularizations, Percutaneous Coronary[Title/Abstract] |
| #71 | OR/59-70 |
| #72 | Risk factor[Title/Abstract] |
| #73 | Risk assessment[Title/Abstract] |
| #74 | Multivariate analysis[Title/Abstract] |
| #75 | Multivariable logistic regression[Title/Abstract] |
| #76 | OR/72-75 |
| #77 | #48 AND #58 AND #71 AND #76 |
